# Bioconductor: Planning a third decade of comprehensive support for genomic data science

**DOI:** 10.1016/j.patter.2025.101319

**Published:** 2025-07-11

**Authors:** Vincent J. Carey

**Affiliations:** 1 Channing Division of Network Medicine, Mass General Brigham, 181 Longwood Avenue, Boston, MA 02115, USA

## Abstract

This opinion piece discusses the Bioconductor project for open-source bioinformatics and the engineering concepts underlying its effectiveness to date. Since the inception of Bioconductor in 2002 with 15 software packages devoted to analysis of DNA microarrays, it has grown into an ecosystem of ∼3,000 packages contributed by more than 1,000 developers. Aspects of the history and commitments are reviewed here to contribute to thinking about the design and orchestration of future open-source software projects.

## Main text

### Introduction

Computational biology has given rise to a number of widely used open-source software ecosystems over the years, including BioPerl, Biopython, Bioconductor, the scverse, and the Seurat toolkit. Tasks addressed include genome browsing and annotation, sequence analysis, and many problems arising in single-cell and spatial omics. However, the economic, social, and technical aspects of open-source ecosystem sustainability for biosciences are not well understood. Resources required for open-source genomic data science change frequently owing to the dynamism of the biotechnological landscape. Growth in volume and complexity of open ecosystem objectives and resources under curation is inevitable given the tendency in the biosciences to strongly prioritize investments in data generation over investments in data management and analysis.^1^

Bioconductor is an open-source software project with the aim to develop and share software packages for precise and repeatable analysis of biological data in R. A core team of developers provides coordination and writes and maintains essential infrastructure and software packages for the project, but most analysis packages are contributed by members of the active user and developer community. All packages distributed by the project are tested regularly on dedicated hardware, referred to as the “Bioconductor Build System.”

This discussion of the Bioconductor ecosystem focuses on engineering commitments that strike a balance between stability required by analysts and dynamism required by investigators and developers at the cutting edge of genome biology. I will focus on features of the project that I believe will ensure Bioconductor’s continued significance to large swaths of research and development efforts in computational biology.

### Software engineering and preparing for change

The Communications of the Association for Computing Machinery recently featured a paper that reviews the special status of software engineering in scientific research.^2^ With the subtitle “Toward RSE research,” in which RSE denotes “Research Software Engineering,” Felderer et al. observe that the development of software for research purposes

(1) has the aim of advancing research fields in a wide variety of scientific domains,
(2) frequently occurs without specification of requirements, and
(3) has verification and validation processes that are “strictly scientific” and often cannot be understood without specific postdoctoral expertise.

The authors remark that “[f]ew scientists are trained in software engineering, which leads to a disregard of most modern software engineering methods.”

When software tools are needed to advance the frontier of knowledge in a given domain, research organizations must strike a balance between speed of delivery required to maintain knowledge leadership and achievement of engineering goals in addition to correctness and verifiability. In a research organization dedicated to knowledge leadership, intellectual and material responses to the complexity of subject matter can easily crowd out thinking and action related to software engineering goals. It is therefore typical for lead investigators of scientific projects to think of engaging programmers rather than engineers. But is it true that much software production for research purposes is done in “disregard of most modern software engineering methods?”

A useful source on current concepts of software engineering is the “flamingo book.^3^” Engineering and programming are compared:

We see three critical differences between programming and software engineering: time, scale, and the trade-offs at play. On a software engineering project, engineers need to be more concerned with the passage of time and the eventual need for change. In a software engineering organization, we need to be more concerned about scale and efficiency, both for the software we produce as well as for the organization that is producing it. Finally, as software engineers, we are asked to make more complex decisions with higher-stakes outcomes, often based on imprecise estimates of time and growth.

What is meant by “the eventual need for change?” Any code artifact may need to change when it is discovered to contain an error or severe inefficiency. An artifact may also need to change because its “environment” (compiler, interpreter, control interface) has changed, some new functionality is desired, or some feature needs to be removed.

### How Bioconductor confronts the “eventual need for change”

Four commitments, established from version 1.0 of Bioconductor 23 years ago, represent engineered solutions to accommodate change.

(1) **Release and devel branches with calendar-based cadence.** The packages in a Bioconductor release are intended to be stable for 6 months. Constraints on feature introduction and removal are detailed in contributor guidelines and a tutorial manuscript.^4^ Bioconductor releases every year follow closely annual upgrades to R. R core developers test new features against the Bioconductor ecosystem, and the Bioconductor devel branch frequently tests against pre-release versions of R. While a new major version of R is released annually, Bioconductor includes a mid-year release to keep pace with changes in applied biotechnology. Bioconductor’s build reports page provides an overview of build/check targets and cadences.
(2) **Dependencies of Bioconductor packages are limited to regulated software repositories.** Package interdependencies are formally declared in package metadata (the DESCRIPTION file) and may only reference packages registered and actively maintained in Bioconductor or CRAN.
(3) **Portability of all packages to all major platforms in use.** Developers must make efforts to ensure that code works on Linux (Intel and ARM chipsets), Windows (Intel), and macOS (Intel and Apple Silicon).
(4) **Continuously tested co-embedded software and documentation.** Manual pages for R functions in packages have optional executable examples. These help users build comprehension through observation of function operation. Executable examples also form an element of verification—code analysis in the R CMD check process includes execution of all example code. The vignette component that is optional for R packages in CRAN is obligatory for Bioconductor packages (and was initially deployed in Bioconductor 1.0). Vignettes are also executable documentation, but typically employ multiple functions and packages, providing verification of interoperability. All examples and vignettes are tested every build cycle—approximately daily for the devel branch, twice weekly for the more stable release branch.

These commitments are real, but limitations and exceptions arise and cause problems. The release cadence is performed with an absolute and irrevocable freeze for code in version x.(y − 1) when version x.y is released. Thus, packages that contain bugs are frozen in Bioconductor’s always-accessible archive of past releases. Allowing dependence on CRAN packages, which do not have a controlled release timetable and do not obey any constraints on feature changes, can lead to widespread testing failures in the event of unanticipated breaking changes to one or more CRAN packages on which Bioconductor packages depend. There are no established methods for identifying a package that is no longer actively maintained, or for finding a maintainer who is able to fix a problem underlying a cascade of failures. Developers of widely reused packages may abandon their code, necessitating intervention by other developers who may have limited experience with the abandoned methods. Faced with growing interest in R-python interoperation, dependence of Bioconductor packages on python packages distributed through PyPI or conda is allowed. This greatly increases the likelihood of broken dependency chains, and acceptable constraints on provenance and version specifications for python dependencies are not fully articulated.

### Unexpected consequences of Bioconductor’s open-source discipline

**Figure 1:**
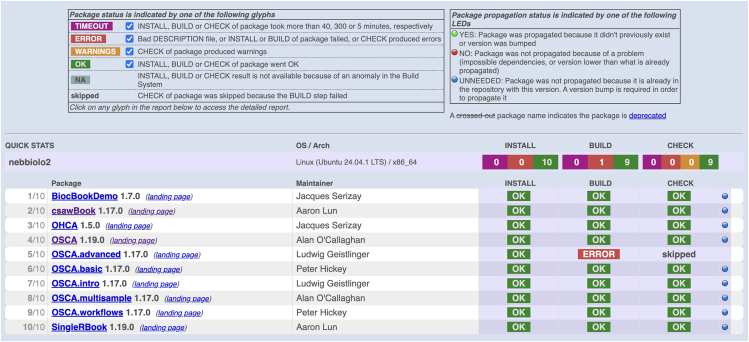
Status of ten continuously tested monograph-scale documents csaw, ChIP-Seq Analysis with Windows; OSCA, Orchestrating Single-Cell Analysis; OHCA, Orchestrating Hi-C Analysis. “SingleR” deals with annotation of cell types in single-cell transcriptomics.

**Figure 2:**
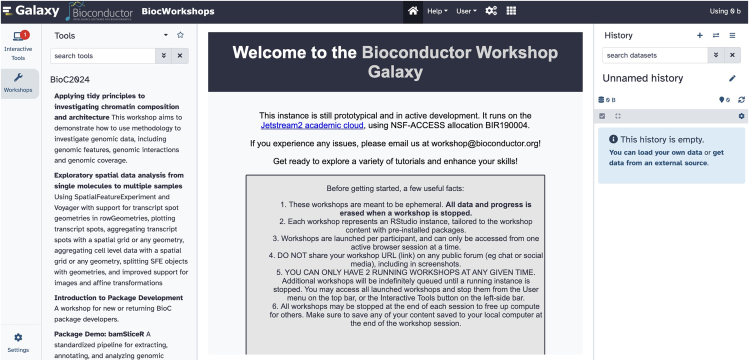
Snapshot of the workshop.bioconductor.org user interface The system produces containers with all necessary software and data and includes authoring support. Scalable computing services handle student activities in RStudio sessions.

With a large and very active user base, demands can come forth that are not easily resisted, even on a fixed budget. Immediately after Apple introduced the M1 chip, users requested precompiled binaries for this platform. Users of Huawei Cloud desired support for ARM Linux, and the Bioconductor Build System was modified to include testing on that platform. More recently, developers can declare that their packages are optionally or necessarily “GPU-reliant”; thanks to support from NHGRI and from the Chan-Zuckerberg Essential Open Source Software program, GPUs are available for integration into the build and test system. Two additional unforeseen obligations are the handling of integrated and continuously tested monograph-scale documents and the creation and maintenance of a system for authoring and deploying multicourse/multiuser workshops. See Figures 1 and 2 for overviews.

### New concepts for decade three

“Keeping the lights on” for a project like Bioconductor is no simple task. Three initiatives that are likely to be undertaken are built and check system renovation, enhancement of “instant-on” and container-oriented genomic data science support, and closer collaboration with the python community on solutions for spatial omics.

*Build system.* Integration of the build and check processes with GitHub workflow automation will take place in 2025 with the collaboration of rOpenSci in the R-Universe project. We aim to preserve the “developer experience” in a way that is satisfactory to all of the hundreds of contributing developers, but we also aim to simplify and standardize the software stack underlying the build and check processes.

*“Instant on” and containerization.* R in WebAssembly facilitates data science in the browser. Most Bioconductor packages have been compiled to WebAssembly so that they can be used in the absence of a full installation of R. This can help expose students to basic aspects of data science in a uniform way and provides avenues toward serverless genomic data science workflows. Bioconductor has long provided Docker containers with R and RStudio. Users of containers need not compile Bioconductor packages from source—all packages can be compiled once for any given container image and then archived to a public repository for rapid installation on request. Furthermore, by using Debian packaging methods, Bioconductor packages can be bound with all system dependencies, allowing successful installation of complex software stacks on containers endowed only with R.

*Social and technical collaboration across ecosystems.* Supported by the Chan-Zuckerberg Essential Open Source Software program, a number of Bioconductor developers attended a SpatialData hackathon in Basel in November 2024. A basic objective is to ensure that users of scverse and Bioconductor can take advantage of the Open Microscopy Environment Next Generation File Format standards for representing spatial omics experiments. This meeting exposed several differences in approaches to developing software and data repositories, and work remains to be done to synchronize interchanges between the ecosystems. A team at Genentech has spearheaded BiocPy, which implements a number of key Bioconductor data structures in python.

### Conclusions

Four aspects of the engineering of Bioconductor allow it to adapt to changes in biotechnology and information science, continually meeting the needs of thousands of researchers around the globe: bifurcated release and devel branches with calendar-based cadence, dependencies constrained to regulated repositories, obligatory portability, and obligatory and continually tested executable documentation. While these practices have been sustainable to date, more difficult engineering and evaluation are likely required for the project to survive another decade. A key deficiency is lack of visibility of actual usage. Download counts and citation counts are favorable and demonstrate strong general impact, but there are no clear metrics at present that would guide infrastructure developers toward bottlenecks, inefficiencies, or even bugs in widely used functions and packages. Test coverage and test rigor are handled informally. So, there is more work to be done, over and above that which can be anticipated to be required as scientific innovation proceeds.

## Acknowledgments

Charlotte Soneson and Levi Waldron provided helpful insights on presentation. This work was supported in part by NIH grant 2U24HG004059-17.

## Declaration of interests

The author declares no competing interests.
